# Tailorable Zinc-Substituted Mesoporous Bioactive Glass/Alginate-Methylcellulose Composite Bioinks

**DOI:** 10.3390/ma14051225

**Published:** 2021-03-05

**Authors:** Vera Guduric, Niall Belton, Richard Frank Richter, Anne Bernhardt, Janina Spangenberg, Chengtie Wu, Anja Lode, Michael Gelinsky

**Affiliations:** 1 Centre for Translational Bone, Joint and Soft Tissue Research, University Hospital Carl Gustav Carus and Faculty of Medicine, Technische Universität Dresden, 01307 Dresden, Germany; vera.guduric@tu-dresden.de (V.G.); nrbelton328@gmail.com (N.B.); richard_frank.richter@tu-dresden.de (R.F.R.); anne.bernhardt@tu-dresden.de (A.B.); JSpangenberg90@aol.com (J.S.); anja.lode@tu-dresden.de (A.L.); 2 State Key Laboratory of High Performance Ceramics and Superfine Microstructure, Shanghai Institute of Ceramics, Chinese Academy of Sciences, Dingxi Road 1295, Shanghai 200050, China; chengtiewu@mail.sic.ac.cn

**Keywords:** mesoporous bioactive glasses, alginate, bioprinting, zinc, ion release

## Abstract

Bioactive glasses have been used for bone regeneration applications thanks to their excellent osteoconductivity, an osteostimulatory effect, and high degradation rate, releasing biologically active ions. Besides these properties, mesoporous bioactive glasses (MBG) are specific for their highly ordered mesoporous channel structure and high specific surface area, making them suitable for drug and growth factor delivery. In the present study, calcium (Ca) (15 mol%) in MBG was partially and fully substituted with zinc (Zn), known for its osteogenic and antimicrobial properties. Different MBG were synthesized, containing 0, 5, 10, or 15 mol% of Zn. Up to 7 wt.% of Zn-containing MBG could be mixed into an alginate-methylcellulose blend (algMC) while maintaining rheological properties suitable for 3D printing of scaffolds with sufficient shape fidelity. The suitability of these composites for bioprinting applications has been demonstrated with immortalized human mesenchymal stem cells. Uptake of Ca and phosphorus (P) (phosphate) ions by composite scaffolds was observed, while the released concentration of Zn^2+^ corresponded to the initial amount of this ion in prepared glasses, suggesting that it can be controlled at the MBG synthesis step. The study introduces a tailorable bioprintable material system suitable for bone tissue engineering applications.

## 1. Introduction

Bioactive glasses are three-dimensional oxide-based compounds (SiO_2_-P_2_O_5_-CaO-Na_2_O) discovered by Hench [1]. Bioactive glasses have been widely used in medical applications for treatment of osseous defects in orthopedics and dentistry since 1985 due to their ability to bond and integrate with the living bone. Their main characteristics are high osteoconductivity, an osteostimulatory effect and high degradation rate, making them promising for bone regeneration applications [2]. Mesoporous bioactive glasses (MBG) are a special type of bioactive glasses, known for their highly ordered mesoporous SiO_2_ channel structure and high specific surface area. Zhu et al. synthesized MBG containing 80, 5, and 15 wt.% of Si, P, and Ca, respectively [3]. This glass showed good cytocompatibility and spreading of human bone-derived cells after seeding on MBG-scaffolds. Thanks to their high specific surface area, MBG channels can be easily loaded with drugs, growth factors, or bioactive ions which can be released later, allowing the use of these glasses as a delivery system [4,5]. Another option for improving the therapeutic activity of the MBG system with bioactive metal ions is to substitute calcium (Ca) in the MBG network with a specific ion-inducing desired therapeutic effect, such as strontium [6,7,8], or copper [9] and cobalt [7]. Zinc has been implemented in dental and orthopedic biomaterials since it has a positive impact on osteoblastogenesis and activity of osteoblasts [10], but it also shows an antimicrobial effect [11,12,13,14]. This ion has been studied for its antimicrobial effect in synergic action with antibiotics [15]. The combination of MBG as particulate biomaterial with other, especially injectable biomaterials broadens its applicability. One interesting and promising option is the integration of MBG into printable biomaterial inks, making it applicable for additive manufacturing techniques [6,16]. Three-dimensional printing, especially extrusion based (bio)printing, is one of the techniques commonly used for fabrication of scaffolds for tissue engineering applications. Extrusion-based (bio)printing allows fabrication of volumetric constructs processing pasty materials and therefore also composites with particulate material. Pure alginate (alg) inks at cytocompatible concentrations have too low viscosity for extrusion-based 3D printing of volumetric scaffolds but Schütz et al. [16] have found that by addition of methylcellulose (MC), the viscosity and shear thinning properties of the resulting algMC blend are strongly enhanced and the blend containing 3% alg and 9% MC allowed printing of volumetric structures, maintaining cell viability [17]. After printing, alginate was crosslinked with calcium ions and MC was released successively, not affecting the macroporous structure [17]. The presence of MC has other advantages such as leaving micropores after being washed out over time, it is biologically inert and does not interact with the crosslinking process [18,19]. The algMC blend was used in past years in our and other groups, as a suitable bioink for printing a variety of cell types such as human mesenchymal stem cells (hMSC) [20], chondrocytes [21,22], rat pancreatic islets [23], and microalgae [24,25]. To achieve further functionalities, this blend was also combined with blood plasma supporting cellular functions or with nanoclay laponite, allowing efficient loading with growth factors like VEGF or BMP-2 [26,27,28]. In the present study, we aimed to combine MBG with the algMC blend to develop an extrudable composite suitable for 3D (bio)printing and delivery of therapeutic metal ions for bone tissue engineering. Due to its positive impact on osteoblastogenesis and activity of osteoblasts, but also its antimicrobial effect, Zn^2+^ was chosen for substitution of MBG added in algMC blend. Bioactive glass/alginate-based hydrogel composites for bioprinting have already been studied, but they contained a low amount of glass up to 1 wt.% only [29,30]. Hence, we investigated various combinations of algMC blends containing a higher amount of MBG than studied by now (up to 7 wt.%) with incorporated Zn^2+^ ions in the glass network. Rheological and printing analyses of composites were conducted, release of Si, P, Ca and Zn ions from the MBG-containing scaffolds was quantified and cell number within bioprinted composite scaffolds were analyzed over cultivation time. This study shows the development and characterization of a tailorable composite bioink which could be used to tailor release of bioactive compounds.

## 2. Materials and Methods

### 2.1. MBG Synthesis

MBG powders (molar ratio of Si/P/Ca = 80:5:15) were synthesized according to the protocol by Zhu et al. [3] and Yan et al. [31]. In brief, 4 g of non-ionic bloc polymer Pluronic (P123, Mw = 5800, Sigma-Aldrich, Steinheim, Germany) was dissolved in 60 g of 96% ethanol and stirred for 1 h at room temperature. Then, 6.7 g of tetraethyl orthosilicate (99%, Sigma-Aldrich, Steinheim, Germany), 0.73 g of triethyl phosphate (99.8%, Sigma-Aldrich, St. Luis, MO, USA), 1.4 g of calcium nitrate tetrahydrate (Merck, Darmstadt, Germany) dissolved in 6 mL of deionized water and 1 g of 0.5 M HCl were added in the solution. After stirring for 24 h, the solution was distributed in Petri dishes for drying at room temperature for one day. Once all of the ethanol evaporated, the obtained gel was dried at 60 °C for 3 h, ground and calcinated in a furnace at a ramp of 2 °C per minute up to 700 °C (7 h). MBG was ground manually to obtain particles smaller than 45 µm, verified by sieving. Zn-modified MBG was synthesized by substitution of Ca by Zn using zinc nitrate hexahydrate (Sigma-Aldrich, Steinheim, Germany) in the respective molar ratios to obtain 5% Zn/10% Ca, 10% Zn/5% Ca or 15% Zn/0% Ca, as shown in Table 1. Zn (NO_3_)_2_•6 H_2_O was dissolved in 25 mL of deionized water due to its lower solubility. All the MBG were gamma-sterilized (25 kGy) prior mixing with algMC blends. Throughout the manuscript, nominal calcium and zinc content in the different ZnMBG is always given in mol%.

### 2.2. Evaluation of the Channel Structure of MBG

Maintenance of mesoporous channel structure in all ZnMBG was investigated with Transmission Electron Microscopy (TEM) using Tecnai TF30 G2 FED-TEM (Thermo Fisher Scientific, Waltham, MA, USA) at 300 kV acceleration voltage with the standard single tilt holder. The images were captured on a Gatan Oneview (Gatan, Pleasanton, CA, USA). Channel size of all the samples was measured by ImageJ.

### 2.3. Preparation of algMC Blends

Alginic acid sodium salt from brown algae (Sigma-Aldrich, Steinheim, Germany) was dissolved at a concentration of 3 wt.% in phosphate buffered saline (PBS) and stirred overnight. The resulting alg solution and MC powder (Sigma-Aldrich, Steinheim, Germany) were autoclaved and stored (alg at 4 °C and MC at room temperature) until ink preparation for 3D printing. To prepare algMC blends, MC powder was added to alg solution to obtain final concentrations of algMC of 3 wt.%: 6 wt.% (later called 3–6 blend) or 3 wt.%: 9 wt.% (later called 3–9 blend). The mixtures were stirred thoroughly with a spatula and left for 30 min to allow the swelling of MC. The resulting blends were then used for printing in their native state or mixed with MBG.

### 2.4. Preparation of algMC-MBG Composite Inks

Composite inks were produced by mixing MBG particles with a spatula into algMC blends under sterile conditions and immediately used for rheological testing or filled into cartridges for printing. For rheological characterization and testing of printability, composites of 3, 5, 7 and 10 wt.% of both 0ZnMBG and 15ZnMBG and additionally of 7 wt.% of 5ZnMBG and 10ZnMBG were produced with both 3–6 and 3–9 blends. Later, composites with 7 wt.% of each ZnMBG were used for bioprinting experiments. Throughout the manuscript, MBG content in the composite inks is always given in wt.%.

### 2.5. Rheological Characterization and Mass Flow Determination

Rheological testing was performed on each ink (algMC 3–6 and 3–9 blends and composites with MBG) using a RHEOTEST RN 4.1 plate rheometer (RHEOTEST Medingen GmbH, Ottendorf-Okrilla, Germany). In this process, 2 g of each ink was placed onto the sample platform. A flat plate probe with a diameter of 36 mm and a plate–plate distance of 500 µm was used to test the viscosity and shear thinning behavior of each ink with increasing shear rate from 0 to 100 s^−1^ over 1200 s.

Mass flow was investigated using the BioScaffolder 3.1 (GeSiM mbH, Radeberg, Germany). Prepared inks were extruded from 10 mL cartridges through conical 410 µm nozzles (Globaco GmbH, Rödermark, Germany) for 10 s at pressures of 50–350 kPa and the mass of the extruded material was measured. Average mass (n = 3) of extruded inks was divided by 10 s to achieve the mass flow rate at each pressure.

### 2.6. Three-Dimensional Printing of Cell-Free Scaffolds and Analysis of Shape Fidelity

Three-dimensional printing of scaffolds was performed using the GeSiM BioScaffolder 3.1. Inks were extruded through conical nozzles with an inner tip diameter of 410 µm, using pressures and printing speeds shown in Table 2. Square shaped scaffolds (width 7.75 mm and strand distance 1.8 mm, seven strands per layer in both directions) consisting of 4 layers were built up in a layer-by-layer fashion, rotating the strand orientation by 90° for each layer. Upon completion of the printing process, each scaffold was placed for 10 min in a 100 mM CaCl_2_ solution for crosslinking of alginate. Then, CaCl_2_ solution was removed and scaffolds were stored in Hank’s Balanced Salt Solution (HBSS) for imaging.

Three scaffolds per ink type were examined concerning their shape fidelity using images of 3D-printed scaffolds obtained with stereo light microscopy (Leica M205C equipped with DFC295 camera, Wetzlar, Germany). Obtained strand widths were compared to the theoretical value representing the extruding nozzle diameter (410 µm).

### 2.7. Cells

Human mesenchymal stem cells (MSC) immortalized by lentiviral gene transfer resulting in expression of human telomerase reverse transcriptase (hTERT) were used [31]. The cell line was provided by Professor Matthias Schieker (Laboratory of Experimental Surgery and Regenerative Medicine, Ludwig-Maximilians-University, Munich, Germany). For cell expansion, Dulbecco’s Modified Eagle’s Medium (DMEM) supplemented with 15% fetal calf serum (FCS; Corning, NY, USA) and 100 U mL^−1^ penicillin/100 µg mL^−1^ streptomycine (1% P/S; Life Technologies, Carlsbad, CA, USA) was used (growth medium). Experimental medium was DMEM supplemented with 10% FCS and 1% P/S.

For observation of cells within scaffolds over time, pre-labelling of cells was performed with Vybrant DiD Cell Staining Solution (Thermo Fisher Scientific, Eugene, OR, USA) prior to mixing into composite inks. Cells were suspended in FCS-free medium at a density of 1 × 10^6^ cells per milliliter. Vybrant DiD stain was added to the suspension in a concentration of 5 µL per 1 mL and incubated in the dark at 37 °C for 25 min, with gentle shaking of the suspension twice during incubation. The cells were then washed twice with 20 mL of FCS-free medium and used for bioink preparation and bioprinting.

### 2.8. 3D Bioprinting

The 3–6 and 3–9 algMC blends and MBG containing composites thereof were prepared in sterile conditions. In this process, 5 × 10^6^ hTERT-MSC (with or without DiD-labelling) suspended in 100 µL of experimental cell culture medium were mixed into 1 g of the ink with a spatula in sterile conditions. GeSiM BioScaffolder 3.1. was used for 3D bioprinting, extruding the bioinks from 10 mL cartridges through conical dosing nozzles with an inner tip diameter of 410 µm. Depending on the bioink composition, pressures of 40–300 kPa were applied as well as different printing speeds of 4–10 mm/s (Table 3) to print cell-laden constructs of the same shape as for the shape fidelity evaluation. Upon completion of the printing process, each specimen was crosslinked for 10 min with 100 mM CaCl_2_ and then cultured in experimental medium at 37 °C and 5% CO_2_ until performing the experiments at defined time points. The cell culture medium was changed every other day.

### 2.9. Cell Number Development over Time within Bioprinted Scaffolds

Cell number development within bioprinted composite constructs (and MBG-free control) was examined by DNA quantification. At each time point (day 3, 7, 14, and 21), cell culture medium was removed, scaffolds were washed twice with PBS and stored at −80 °C until analysis. To extract DNA from cell-laden scaffolds they were thawed at room temperature and 3 mL of sodium citrate (100 mM) was added to each well. The well plates were put on a shaker for 3 h while the scaffolds dissolved; the suspensions were then transferred to 15 mL centrifugation tubes for overnight lysis in a 60 °C water bath. The samples were then sonicated on ice for 10 min, frozen and then thawed again to get rid of MC, while the MBG particles stayed on the bottom of the tubes. DNA quantification was performed using the QuantiFluor dsDNA system (Promega, Madison, WI, USA) as per the manufacturer’s instructions and measured on a microplate reader (Infinite 200 PRO, Tecan, Switzerland). Excitation and emission wavelengths were 485 and 535 nm, respectively.

Calibration line to relate amount of DNA detected to number of cells was prepared using aliquots of defined hTERT-MSC numbers in 1.5 mL tubes. After freezing and thawing, the cell pellets were treated with 250 µL of sodium citrate (100 mM), followed by DNA extraction and quantification as described above for the bioprinted composite constructs.

### 2.10. Ion Release

Bioprinted constructs were used for the ion release experiment. The cell culture medium (2 mL) was collected every time it was changed. The same experiment was performed on MBG bulk powders as well. The mass of the bulk MBG samples was the same as the MBG content in one scaffold (approximately 0.017 g) and the same volume of cell culture medium (2 mL) was used, in order to compare ion release from bulk MBG and from printed composites. All the collected media were stored at 4 °C until analysis. Released concentrations of Si, P, Ca, and Zn ions were analyzed using inductively coupled plasma-optical emission spectroscopy (ICP-OES, Plasma Quant PQ 9000 Elite, Analytik Jena, Jena, Germany). Samples were diluted with 2% nitric acid (1:5) prior to the experiment. Experimental medium was analyzed as well and detected concentrations of selected ions were deducted from test samples.

### 2.11. Statistics

Two-way analysis of variance (ANOVA) with Dunnett’s multiple comparison in GraphPad Prism 8 was performed to determine statistical significance of differences of obtained data of print fidelity and cell number development. One-way ANOVA with Tukey’s multiple comparison was used to analyze data of the ion release study.

## 3. Results

### 3.1. Observation of Mesoporous Structure in ZnMBG

**Figure 1:**
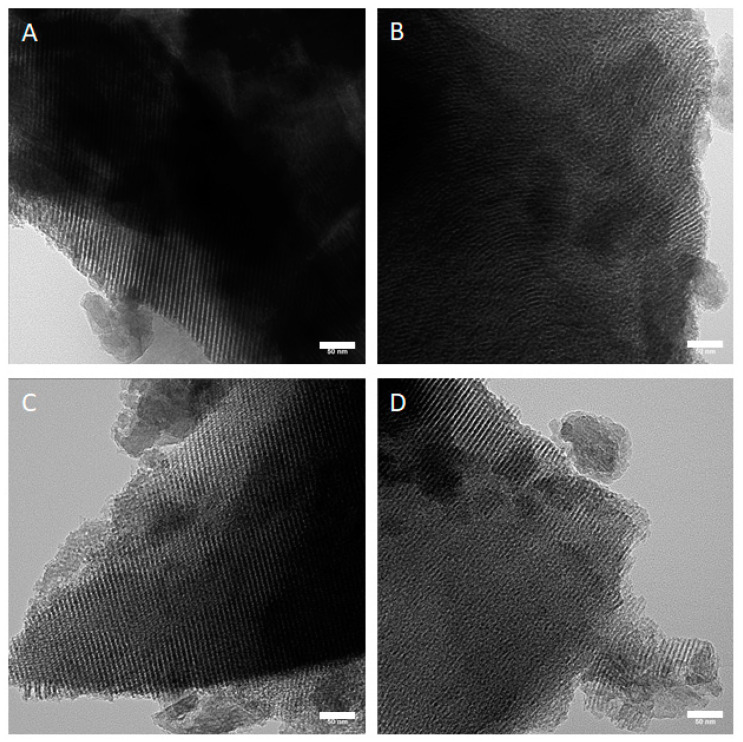
TEM images of synthesized MBG showing channel structures in all samples. (**A**) 0ZnMBG, (**B**) 5ZnMBG, (**C**) 10ZnMBG and (**D**) 15ZnMBG. Scale bars represent 50 nm.

Since the (partial) substitution of Ca by Zn can modify the mesoporous structure of MBG, TEM analysis was performed to observe the structure in all MBG samples. Parallel aligned channels could be observed in all the analyzed particles. Representative images are shown in Figure 1. Channels in all the particles were approximately 7 nm in width.

### 3.2. Rheological Characterization and Analysis of Mass Flow

**Figure 2:**
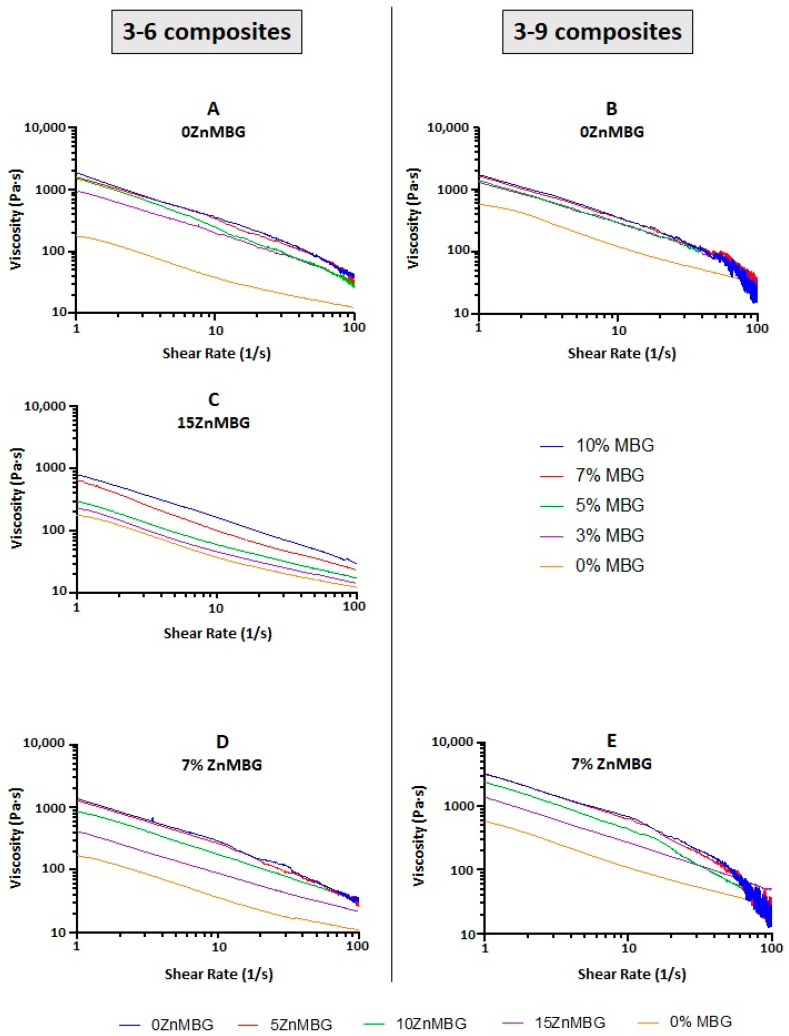
Rheological analysis of composite inks. Representative curves out of 3 measurements for each composition: (**A**) 3–6 algMC blend and (**B**) 3–9 algMC blend with different amounts of basic MBG containing 15% Ca and no Zn (0ZnMBG), (**C**) 3–6 algMC blend with different amounts of MBG containing 15% Zn and no Ca (15ZnMBG), (**D**) 3–6 algMC blend and (**E**) 3–9 algMC blend with 7% of MBG containing different amounts of Zn (0, 5, 10 and 15%) and Ca (15, 10, 5, 0%).

In order to investigate how the integration of MBG affects the rheological properties of algMC blends, different amounts (0, 3, 5, 7 and 10%) of 0ZnMBG (basic MBG containing 15% Ca) were added into both, 3–6 and 3–9 blends. The plots of both the 3–6- and 3–9- based composites with 0ZnMBG show that viscosities of all the composites containing 3–10% MBG were closer together, while the MBG-free control showed a clearly lower viscosity (Figure 2A,B). Increasing the amount of added MBG induced an increase in the resulting composite’s viscosity, making mixing of the glass particles and their even distribution within the inks more difficult, especially in the case of the 3–9 blend.

Since it was more practical to mix MBG with the 3–6 blend due to its lower viscosity and suitable rheological and printing properties were achieved with its composites, the same analysis was performed with composites prepared using 3–6 blend and different amounts of 15ZnMBG. The aim was to investigate if the presence of Zn in MBG instead of Ca changes the rheological properties of the resulting composites. Figure 2C similarly shows a direct correlation between viscosity of the 3–6 ink with 15ZnMBG and amount of MBG in the composite, except that the values seem to be evenly distributed from 0% MBG up to 10% MBG. The viscosity for 10% MBG appears to be one order of magnitude lower for composite with 15ZnMBG (0% Ca) than with 0ZnMBG (15% Ca).

Since the addition of 7% of these MBG seemed to be the most appropriate concentration for all the composite inks regarding mixing, rheological and later printing properties, both algMC blends (3–6 and 3–9) were combined with 7% of each ZnMBG containing different amounts of Zn (0, 5, 10, and 15 mol-%). Figure 2D,E shows increasing viscosity of both, the 3–6 and 3–9, inks containing 7% of each ZnMBG, with decreasing amount of Zn (i.e., increased amount of Ca) in the ZnMBG. Viscosities of the 3–9-based composites with 7% of each ZnMBG were grouped closer together, with a larger disparity between the viscosities of control ink without any MBG and composites with 7% of 15ZnMBG.

**Figure 3:**
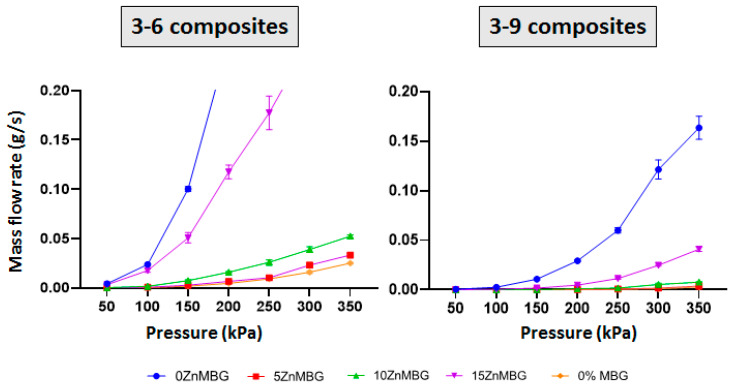
Mass flow analysis of 3–6 and 3–9 composite inks containing 7% of each ZnMBG as well as control inks without MBG at increasing pressures from 50 up to 350 kPa. Values on the plots are means ± SD, n = 3.

Mass flow was measured for 3–6- and 3–9-based composite inks with 7% of each ZnMBG and control inks without any MBG (Figure 3). The plots show an increase in the slope of each curve at each consecutive pressure starting from 50 kPa. No material was extruded at 50 kPa with the 3–6 composites containing 0ZnMBG and 5ZnMBG. Higher amounts of Zn in the glass show more distinct increases in line slope. In case of the 3–9 blend, no material was extruded with pressure below 150 kPa for composites containing 10ZnMBG and below 200 kPa for those containing no Zn or the lowest amount of this ion (0ZnMBG and 5ZnMBG). Mass flows for all 3–9 composite inks were lower than for the 3–6 composites with corresponding ZnMBG.

### 3.3. Three-Dimensional Printing of Cell-Free Scaffolds and Shape Fidelity

**Figure 4:**
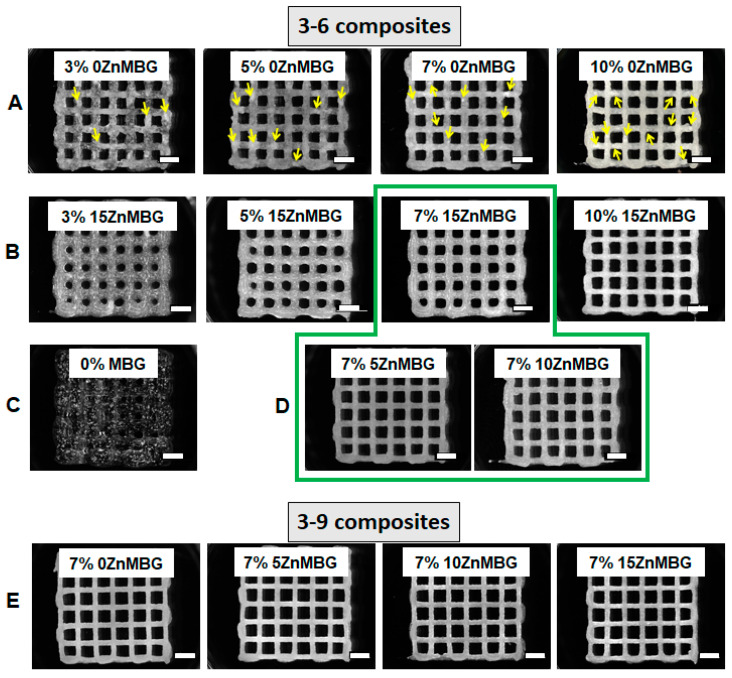
Stereo-light microscopic images of 3D scaffolds printed using different inks. (**A**) The 3–6 composite inks containing 3, 5, 7 and 10% of 0ZnMBG (15% Ca), yellow arrows show agglomerates of added MBG, (**B**) 3–6 composite inks containing 3, 5, 7 and 10% 15ZnMBG (0% Ca), (**C**) 3–6 ink without any MBG, (**D**) 3–6 composite inks containing 7% of 5ZnMBG (10% Ca) and 7% of 10ZnMBG (5% Ca). Green line marks selected combinations of 3–6 composite inks for further experiments, (**E**) 3–9 composite inks containing 7% of each ZnMBG. Scale bars represent 2 mm.

Scaffolds were printed at first with 3–6 composite inks containing 0, 3, 5, 7 and 10% of 0ZnMBG or 15ZnMBG and imaged with a stereomicroscope directly after printing and before crosslinking (Figure 4A,B). The low viscosity of the 3–6 blend without any MBG was not sufficient to print scaffolds with stable macropores; strands were collapsing shortly after extrusion, inducing closing of pores before finalization of the printing process (Figure 4C). Composites with 0ZnMBG did not mix very uniformly, as agglomerates of MBG formed in the ink, which could be seen easily in the printed scaffolds (yellow arrows in Figure 4A), sometimes clogged the nozzle during printing. Inks with increased amount of Zn in the ZnMBG mixed into algMC blend more uniformly and produced scaffolds that had smoother, glossier strands, but whose pores were more likely to collapse. Due to inks’ low viscosities, pores of scaffolds containing 3, 5, and 7% of 15ZnMBG were quickly collapsing and they had to be imaged or crosslinked immediately after printing (Figure 4B). Since mixing and printing of composites was the most appropriate when 7% of MBG was added, scaffolds of the same shape were also printed using both 3–6 (green line marking composite scaffolds in Figure 4D) and 3–9 (Figure 4E) inks containing 7% of glass with different amounts of Zn. Strands of scaffolds printed with 3–9 ink containing 7% of each ZnMBG did not collapse immediately and scaffolds retained the macropores throughout the printing process (Figure 4E). The composite of 3–6 ink with 7% 15ZnMBG showed the lowest viscosity and could only be printed to a height of 8 layers (approx. 2 mm) before either pores collapsed or the z-offset of the extrusion nozzle did not match the current layer height. In contrast, all the other composites containing 7% of each ZnMBG could be printed at least to a height of 20 layers (approx. 5 mm).

**Figure 5:**
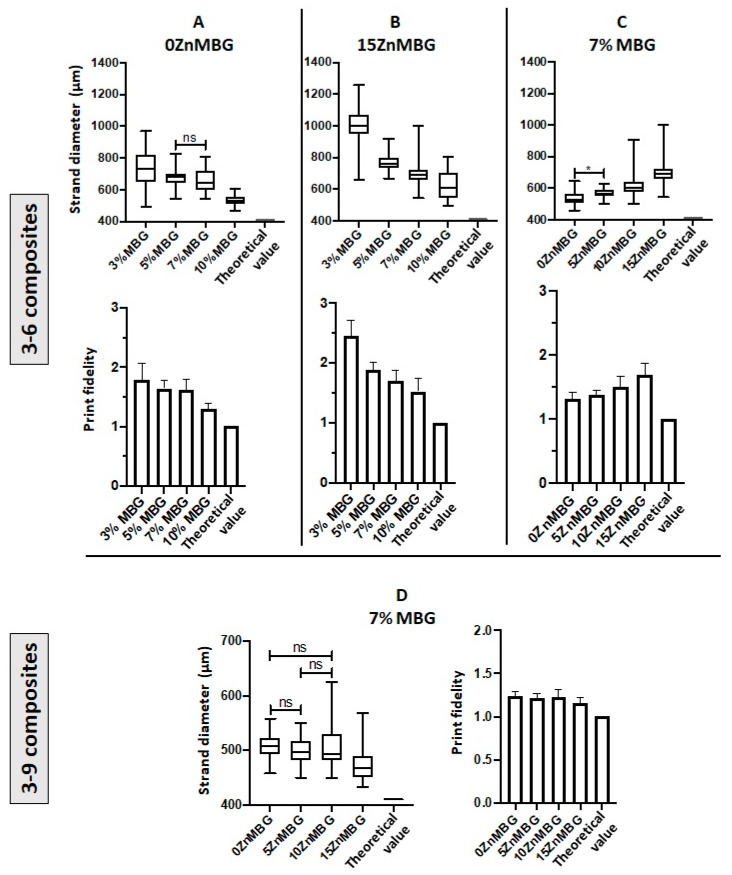
Strand diameters and print fidelities of 3–6 and 3–9 composite scaffolds. (**A**) Strand diameters (upper) and print fidelity (lower graph) of scaffolds printed with 3–6 composites containing different amounts of basic MBG without incorporated Zn (15% Ca). (**B**) Strand diameters (upper) and print fidelity (lower graph) of scaffolds printed with 3–6 composites containing different amounts of 15ZnMBG (0% Ca). (**C**) Strand diameters (upper) and print fidelity (lower graph) of scaffolds printed with 3–6 composites containing 7% of each ZnMBG. (**D**) Strand diameters (left) and print fidelity (right) of scaffolds printed with 3–9 composites containing 7% of each ZnMBG. Value 1 in the shape fidelity charts corresponds to the inner diameter of the extrusion nozzle of 410 µm (theoretical optimum). All strand diameters are significantly different from the theoretical optimum within each graph with *p* < 0.0001, except for *, which has *p* < 0.05, and “ns”, which are not significant; Values are means ± SD, n = 3.

Averaged strand diameters measured on images of non-crosslinked scaffolds directly after printing are shown in Figure 5. Composites with higher amounts of MBG, with or without Zn, could be printed with smaller strand diameters; by increasing the amount of 0ZnMBG and 15ZnMBG in 3–6 composites, significantly different strand diameters were achieved, with the exception of 5 vs. 7% 0ZnMBG (Figure 5A,B). In addition, increased amounts of MBG in the composites produced scaffolds with shape fidelities closer to that of the control (Figure 5A,B). However, with an increasing amount of Zn (i.e., decreasing amount of Ca) in the ZnMBG in composites, printed scaffolds had significantly different strand diameters that diverged further from the control value of 410 µm and decreasing shape fidelity (Figure 5C). Thus, higher amounts of MBG in the composites and lower amounts of Zn in the MBG resulted in the highest print fidelities (sum of strand diameter and shape fidelity). In the case of 3–9 composites with 7% of ZnMBG, average strand diameters shown in Figure 5D were not significantly different from each other, with the exception of the 15ZnMBG composite, in which they were significantly lower. Additionally, they showed a general decrease in strand diameter corresponding to an increase in Zn in the ZnMBG. The shape fidelities of each 3–9 composite were very similar and closer to theoretical value, compared to 3–6 composites.

### 3.4. Cell Number within Bioprinted Composite Scaffolds

**Figure 6:**
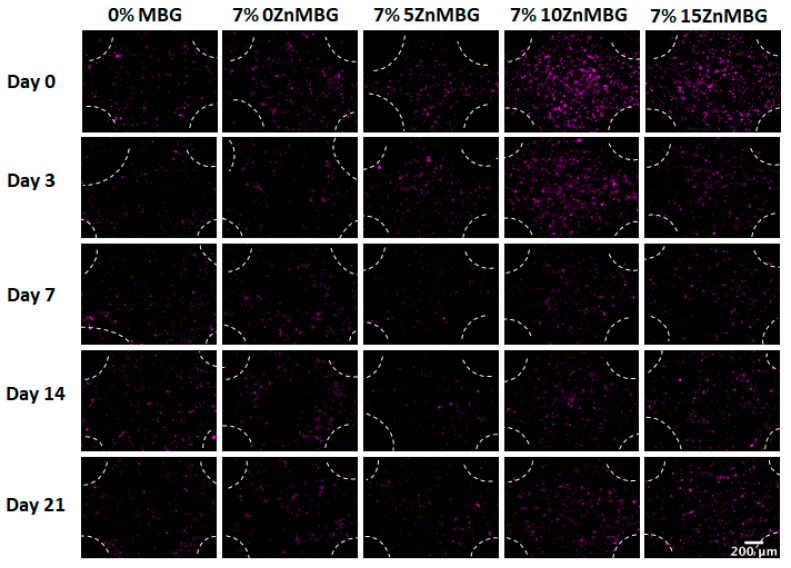
Fluorescence-microscopic images of Vibrant DiD pre-labeled MSC within bioprinted 3–9 composite scaffolds containing 7% of each ZnMBG and 3–9 algMC blend (0% MBG) during 21 days of culture. All images are taken at crossings of printed strands. White markings show the borders of printed strands and pores. Scale bar represents 200 µm and the cells appear as violet spots.

The first bioprinting experiment was performed with 3–6 composites (data not shown), but the addition of 100 µL of cell suspension per gram of the ink highly affected viscosities of inks and decreased their printability but also the stability of the crosslinked constructs in cell culture medium over time. Scaffolds containing 10ZnMBG and 15ZnMBG (5 and 0% Ca, respectively) were stable for the entire duration of the experiment, but those containing 0ZnMBG and 5ZnMBG (15 and 10% Ca, respectively) were falling apart by day 14. Even though 0ZnMBG 3–6 had good printability, scaffolds showed low stability during culture. Therefore, bioprinting experiments were performed only with 3–9 composites. Addition of cell suspension to 3–9 composite inks induced a decrease in viscosity as well, but print fidelity was not affected. Vybrant DiD pre-relabeled cells encapsulated in composite scaffolds were observed immediately after printing and after 3, 7, 14 and 21 days of culture of the printed and crosslinked scaffolds (Figure 6). It appears as if more cells were present in the case of higher Zn amount (10ZnMBG and 15ZnMBG) right after printing (day 0), but their number decreased over time.

**Figure 7:**
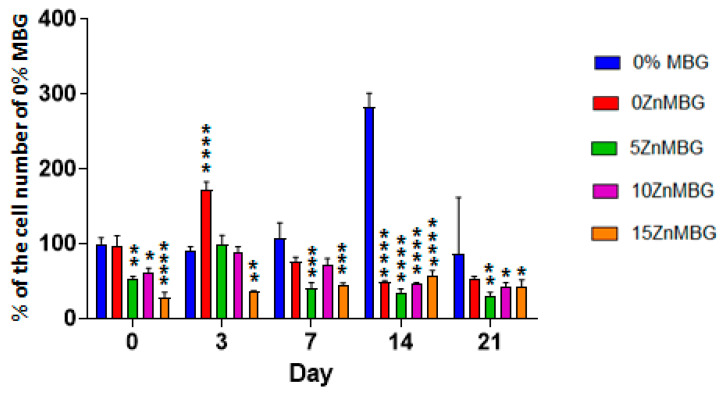
Development of number of MSC in bioprinted 3–9 composite scaffolds containing 7% of each ZnMBG and 3–9 algMC (MBG-free) scaffolds during 21 days of culture examined by quantification of DNA extracted from cells encapsulated in bioprinted constructs. Cell number development is represented as percentage of MBG-free (control) samples right after printing (day 0), * indicates significant difference with *p* < 0.05, ** with *p* < 0.01, *** with *p* < 0.001 and **** with *p* < 0.0001, in comparison to MBG-free constructs; Values are means ± SD, n = 3.

Determination of cell number was performed by DNA quantification using composite scaffolds and MBG-free control. Cell number development over time is shown in Figure 7 as a percentage of the number of cells of MBG-free control at day 0 (right after printing). Cell numbers stayed stable in scaffolds printed with 3–9 algMC bioink without any MBG during 14 days and then sharply increased, while all composite scaffolds contained significantly less cells (*p* < 0.0001). Directly and three days after printing, the cell numbers in composite scaffolds containing Zn-modified MBG was significantly lower than those in composite scaffolds containing 0ZnMBG; at later cultivation time points (days 7, 14, 21), this effect of Zn-modification was not observed.

### 3.5. Ion Release from algMC-MBG Scaffolds and MBG

**Figure 8:**
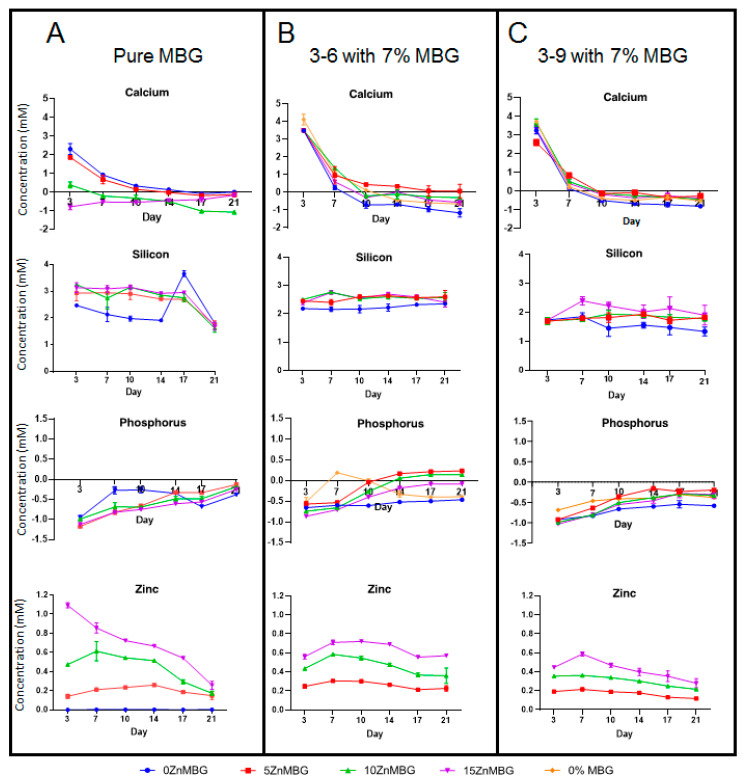
Release of different ions (**A**) from bulk MBG and (**B**) from cell-laden 3–6 and (**C**) 3–9 composite scaffolds containing 7% of each ZnMBG during 21 days; since no Zn was released from 0ZnMBG (blue line) and from 0% MBG (orange line) samples (data not shown), these values are not presented in (**B**,**C**). Values for released concentrations of Si from 0% MBG are not presented in (**B**,**C**) for the same reason. Values are means ± SD n = 3.

The released concentrations of ionic species present in MBG (Si, P, Ca and Zn) from the bioprinted (cell-laden) scaffolds and—for comparison—from bulk MBG were observed over a period of 21 days (Figure 8). In the case of calcium and phosphorus, concentrations are normalized to the concentrations in cell culture medium (releasing medium) representing 0 on the charts.

The released concentration of silicon (as siliceous ions) was stable over 14 days for 0ZnMBG and over 17 days for all Zn-modified MBG and it sharply decreased at the day 21 (Figure 8A). Meanwhile, 3–6 and 3–9 composite scaffolds released Si in stable manner over 21 days with measured concentration of slightly above 2 mM for 3–6 composites (Figure 8B) and slightly less than 2 mM for 3–9 composites (Figure 8C).

An uptake of P (as phosphate) was observed with all tested samples. This uptake was decreasing over time, but it was observed during the entire experiment with bulk MBG. 0ZnMBG showed the lowest affinity for taking up the phosphate ions while all Zn containing MBG showed similar tendency (Figure 8A). In the case of composites, this uptake was slightly stronger with 3–9 based scaffolds than with 3–6 (Figure 8C). Only the 3–6 composites containing 5ZnMBG and 10ZnMBG showed very low release of P after 14 days and the 3–6 MBG-free scaffolds releasing low concentration of this element at the day 7 (Figure 8B).

Bulk glasses containing Ca show initial release of Ca^2+^ at concentration around 1.5 mM, which then drops gradually by day 21 (Figure 8A), where 0 mM corresponds to the concentration in the control medium. All 3–6 and 3–9 composite scaffolds (Figure 8B,C) showed high release of Ca^2+^ with concentrations around 3–4 mM on the first day of media collection, including MBG-free scaffolds, with slightly higher release in the case of 3–6 samples. However, all concentrations of released Ca^2+^ dropped sharply after the first day, showing an uptake of calcium after 7 days, which stayed stable up to day 21. As expected, highest Ca^2+^ concentration was released from the bulk samples containing 15% and 10% of Ca (0 and 5% Zn, respectively) after 3 days. Over time, a decreasing amount of released Ca^2+^ from bulk MBG samples containing more Zn was observed in all the cases except for the 15ZnMBG (0% Ca), as expected (Figure 8A). On the other hand, constant uptake of Ca^2+^ was observed for all the 3–6 composite samples from day 7 and for 3–9 composites from day 14 onwards. There was no significant difference in ion release between bulk MBG samples over time. The same was for release of Ca and P for all the composite samples. On the other hand, significant differences were observed in the release of Si between 0ZnMBG and 5ZnMBG 3–6-composites (*p* < 0.0001), between 0ZnMBG and 10ZnMBG *(p* < 0.05) and between 0ZnMBG and 15ZnMBG composites with 3–9 blend (*p* < 0.0001). All the composite scaffolds were significantly different with each other in the release of Zn (*p* < 0.0001).

All bulk and composite samples released similar respective concentrations of zinc after 3 days. After that, released concentration of this ion from bulk glasses was gradually decreasing by day 21, while an increase was observed at day 3 in the case of 3–6 and 3–9 printed scaffolds. The release of Zn^2+^ from composite scaffolds seems to be constant over time with higher released concentrations corresponding to the higher initial amount of this ion in the samples, without any significant difference between 3–6 and 3–9 composites (Figure 8B,C).

## 4. Discussion

Bioactive glasses are often used for the treatment of osseous defects in dental and orthopedic surgery thanks to their osteoconductivity, osteostimulatory effect and fast degradation [32]. Besides these outstanding properties, mesoporous bioactive glasses (MBG) possess highly ordered Si-based channel structures with high specific surface areas [33], making them especially suitable for growth factor, drug or bioactive ion delivery. MBG could be mixed with different pasty materials to broad its applications. For example, Kauschke et al. prepared an injectable composite ink by mixing brain-derived neutrophic factor-loaded MBG and a pasty α-tricalcium phosphate-based hydroxyapatite-forming bone cement for filling of bone defects [34]. In other studies, MBG was mixed with poly(vinyl alcohol) or with the pasty calcium phosphate cement to make it suitable for 3D printing [6,35]. Moreover, MBG networks can be modified by substitution with different bioactive ions, which could be released during the glass degradation process. In this study, Zn was chosen, which stimulates osteoblastogenesis and shows antimicrobial effect [12,36]. In another study, 5 mol% Zn-modified MBG nanoparticles with hBMSC showed good cytocompatibility and upregulated expression of alkaline phosphatase (ALP) and bone sialoprotein [37]. MBG containing 8 mol% of Zn improved the differentiation of osteoblast-like cells (MG-63) and demonstrated a higher ability to adsorb proteins, in comparison to MBG without Zn [38]. Small amount of this ion (0.4 and 2%) was successfully integrated in MBG network and then mixed with polycaprolactone (PCL) to make it 3D printable [39]. Since produced scaffolds had to undergo high temperatures to get rid of PCL, they were not suitable for bioprinting with cells.

On the other hand, alginate-based hydrogels have been commonly used for bioprinting of cells and they can be easily modified to tailor their final properties, e.g., by combination with different biomaterials, albeit it is sometimes difficult to overcome the challenges regarding printability of the modified inks, mechanical properties and cell response within the bioprinted constructs [40]. Hence, our aim here was to develop a bioink suitable for cell printing containing MBG with varying amount of Zn. Modifying already known MBG (0ZnMBG containing 15 mol% Ca), three additional MBG were successfully synthesized: 5ZnMBG (5 mol% Zn and 10 mol% Ca), 10ZnMBG (10 mol% Zn and 5 mol% Ca) and 15ZnMBG (15 mol% Zn and no Ca). Knowing that there was no phase separation step during the MBG synthesis, there was no loss in any of added ions by the end of the process. Different studies dealing with MBG synthesized in the same or similar way, having the same basic glass architecture (80% Si and 5% P), did not observe significant difference between nominal and actual composition of synthesized MBG [38,41,42]. Substitution of Ca with Zn did not affect the structure of MBG as all synthesized MBG containing Zn maintained mesoscopic pore channel structure of the same size.

Starting with the 3–9 algMC blend which showed promising results in previous work by our group as a versatile bioink for bioprinting of different cell types [17], we added to this ink different amounts of MBG particles (3, 5, 7 or 10 wt.%), which was much more than the amount of added bioactive glass in previous studies of alginate-based composites, dealing with amounts up to 1 wt.%. In one of them, the addition of 0.1 and 0.5 wt.% of bioactive glass into alginate dialdehyde-gelatin ink did not affect viability of MG-63 cells in printed scaffolds [29]. In another study, 1 wt.% of bioactive glass in gelatin-alginate ink slightly decreased viability and proliferation of hMSC in correlation to the increased viscosity induced by the addition of the glass, but stimulated early osteogenic differentiation [30]. With the higher amount added in our study, even higher viscosities of the inks were expected. Therefore, in addition to established 3–9 algMC ink, we investigated the ink containing only 6 wt.% MC (3–6 algMC ink) as well. Inclusion of MBG containing 15 mol-% Ca (0ZnMBG) in both the 3–9 and 3–6 blend greatly increased the viscosity of the inks and required higher printing pressures (Figure 2). Similar effect on the viscosity was observed in another study where MBG particles were added to sodium alginate [43]. Higher viscosity can result from a higher solid content due to the addition of particulate material (in our case MBG particles). The same effect was observed in the case of magnetite microparticle-containing algMC inks, where with an increased amount of these particles the overall viscosity was elevated, resulting in solid state of the ink when more than 50% of particles was added, making it not printable [44]. A similar effect was observed with lower amounts of glass (0.1 and 0.5%) where crosslinking time was shorter when more glass was added [29]. Additionally, higher viscosity can be caused by a pre-crosslinking effect due to sudden release of calcium ions from the MBG.

Due to the different MC amount, the viscosities of the 3–9 composites were higher compared to those of the 3–6 composites. Accordingly, mass flow rates of 3–9 composites at tested applied pressures were lower than corresponding ones of the 3–6 composites and the impact of the added MBG particles was stronger, as shown in Figure 3. Additionally, it was more difficult to uniformly mix MBG into the 3–9 blend. Therefore, 3–6 blend was chosen as the preferable one, combined with 7 wt.% of MBG. These findings suggest that it is possible to increase the amount of MBG particles in algMC blends with the respective decrease in the MC content, allowing to tailor already established (bio)inks described in the literature.

When calcium in MBG was substituted with divalent ion Zn^2+^, also known as a crosslinker of alginate but weaker than Ca^2+^, a pre-crosslinking effect was expected, too [45]. The viscosities of the 15ZnMBG-composites were lower than those of 0ZnMBG-composites and a more gradual increase in viscosity was revealed with increasing 15ZnMBG content than in case of 0ZnMBG. This suggested that Zn^2+^ ions did not contribute greatly to pre-crosslinking of alg, as already explained by Chan et al., confirming that Zn^2+^ has a weaker effect on crosslinking of alginate than Ca^2+^ [45].

Comparing the MBG with different amounts of Zn, it was more difficult to uniformly mix 7 wt.% of ZnMBG with higher amounts of Ca into algMC blend, especially into already more viscous 3–9 blend. This could be explained by the apparent pre-crosslinking effect of Ca: Sometimes, the formation of aggregates was observed in these highly viscous composite inks, probably due to local alginate network formation that hampers movement and therefore distribution of the particles. These aggregates would sometimes clog the nozzle during the printing process. However, all composites (3–6 and 3–9) containing different ZnMBG could be finally printed with good shape fidelity. On the other hand, when cell suspension was added to the 3–6 composite inks (100 µl/g of ink), a notable decrease in viscosity was observed, requiring much lower pressure and higher speed to print scaffolds with defined macropores. This effect was observed for all composites containing MBG, especially for those with higher amount of Zn (less Ca). The viscosities of 3–6 composites were very low in the first bioprinting experiment; therefore, the second one was performed with 3–9 ink, assuming that the addition of cell suspension would have a similar effect on viscosity. Unlike the 3–6 ink, the 3–9 ink showed little difference in viscosity between composites with the different ZnMBG types (data not shown) and it was possible to bioprint these composites with acceptable shape fidelity (Figure 4 and Figure 5). The stability of bioprinted constructs over time was depending on the amount of Zn and therefore Ca in MBG added in the algMC blend. Contradictory to the fact that Ca^2+^ as stronger crosslinker of alginate than Zn^2+^ provides higher stability of the crosslinked hydrogel [46], 15ZnMBG (15% Zn/0% Ca in MBG) composite scaffolds were more stable in cell culture medium over time than those containing 0ZnMBG (0% Zn/15% Ca). It might be possible that composite scaffolds containing low amounts of Zn (0ZnMBG and 5ZnMBG) degraded faster, knowing that Ca-containing MBG degrades quickly [47]. Furthermore, increased presence of Zn in bioactive glasses has a direct effect on their degradation rates [48]. Knowing that the stability of implants and their durability are important in bone tissue engineering, our findings suggest that composite constructs containing MBG with higher amount of Zn than Ca would be probably more stable after being implanted. On the other hand, such hydrogel-based composite materials would not have suitable mechanical properties to be implanted in load-bearing locations.

The live/dead cell viability assay or other assays based on fluorescent signals of cells are very common methods to evaluate cell responses when they are seeded on scaffolds or bioprinted together with hydrogels. While there are some groups evaluating cell viability in bioprinted scaffolds containing bioactive glass particles [29,49], there is a lack of publications presenting similar results with MBG containing scaffolds and cells. The reason for this is that MBG can adsorb metabolic staining and fluorescent dyes and show a signal in various fluorescent channels, making identification of cells difficult. From our own experience, both cells and MBG particles can emit fluorescence signals. Knowing that the MBG particles used in our study were smaller than 45 µm, it would not be possible to distinguish them from cells. Therefore, we decided to prelabel hMSC with Vybrant DiD prior to mixing into the composites and we could easily distinguish them from MBG particles during fluorescence imaging, as shown in Figure 6. We observed an increase in stained cells in MBG-free biopolymer blend constructs, while the cell number in ZnMBG containing composite scaffolds did not increase over time. Similar results were reported by Ojansivu et al., performing live/dead staining of SaOs-2 cells in alginate bioinks with and without bioactive glass. [30]. To supplement these qualitative data, quantification of DNA extracted from cells bioprinted with 3–9 composites was performed. Even though cell number in all ZnMBG containing constructs was significantly lower right after printing, it increased in 0ZnMBG samples after 3 days, indicating that the addition of MBG had a positive effect on the cells. This result is in correlation with results of Du et al. who observed a positive effect of MBG on human bone MSC cell proliferation within bioprinted scaffolds during one week of culture [50]. On the other hand, we observed a decrease in cell number at the following time point and then it stayed stable, but lower than in MBG-free constructs, until the end of the experiment, compared to the cell number in MBG-free constructs right after printing. A decrease in cell number in all the samples, including MBG-free ones, was observed at the end of the experiments. After 21 days, cell number in bioprinted 15ZnMBG composites was similar to 0ZnMBG and MBG-free samples (around 100% in respect of MBG-free samples right after printing), without any significant difference with control (0% MBG). This suggests that non-substituted (0ZnMBG) and fully Zn-substituted MBG (15ZnMBG) did not have a negative effect on cell number development over time in bioprinted constructs. However, cell number was lower in Zn-containing composite constructs at the beginning of the experiment, with an increase over time, comparing to 0ZnMBG samples (Figure 7). The reason for this is probably the higher Zn concentration, which was released shortly after printing which might have had a negative effect on the cells. A similar effect was observed when a higher concentration (0.5 mM) of Zn^2+^ solution induced low viability of MSC [51]. In that study, an aqueous ZnSO_4_ solution was added in cell culture medium. Since our system contains other ions than only Zn^2+^, such as silicates and phosphates and sometimes Ca^2+^, it is possible that they would have a beneficial effect on cells, counteracting the cytotoxic effect of Zn^2+^ ions. Bioprinted composites were not completely stable in cell culture medium over time and their degradation was obvious by the end of the experiment, meaning that cells were probably washed out with the degraded part of the material, explaining the decrease in cell numbers. Cell attachment onto composite scaffolds was not investigated in the present study and it could be an interesting point for a future research to evaluate the effect of MBG presence on cell attachment rate.

Besides the importance of different ion content on stability of the scaffolds, the concentration of released Zn^2+^ and Ca^2+^ ions was of interest in the present study for the possible biological effects of these ions. Therefore, release of all ions from 3–6 and 3–9 composite scaffolds in cell culture medium over time was examined in detail (Figure 8). It appeared that more Ca^2+^ was initially released from composite scaffolds than from bulk glasses, suggesting that algMC has an influence on ion release or that was remaining Ca^2+^ from the crosslinking reaction. While the release of calcium ions from bulk glasses appeared to be constant over time, first a higher release and later an uptake of this ion by composite scaffolds was observed. The reason for this observation could be that alginate was taking up Ca^2+^ needed for later stability of printed constructs, knowing that the stability was decreasing over time. This has been already explained by ionically crosslinked alginate releasing divalent ions and therefore dissolving in physiological conditions [52]. Another explanation might be the formation of hydroxyapatite layer, which had already been observed for gelatin-alginate bioinks [30,53]. It would explain a constant uptake of phosphorous (phosphate) observed with both types of composites. It was found in another study that hydroxy-carbonate-apatite layer was formed on the bioactive glass surface after 12 h in simulated body fluid [54]. Another group observed this effect already after 5 min [55], which would explain the reduced viscosity when cell culture medium was mixed into composite bioinks, taking up the calcium by serum proteins which could induce the already described pre-crosslinking effect. Another reason for an uptake of phosphate could be the formation of other phases than hydroxyapatite. It has been shown that in the presence of Zn^2+^ the growth of hydroxyapatite crystals could be inhibited [56]. In this case a bioactive hopeite-like structure (Zn_3_(PO_4_)_2_·4H_2_O) could be precipitated on the surface of hydroxyapatite, highly depending on pH and Zn^2+^ concentration [57]. Depending on the concentration of Zn^2+^, it can be also substituted into the hydroxyapatite crystal lattice, revealing bioactive and antimicrobial activity against Staphylococcus aureus and Escherichia coli [58,59]. It was demonstrated that when cells were exposed to this environment, the biomineralization started with Zn-hydroxyapatite nucleation within the cell [60]. Taken together, previous studies suggest that no potential problems of incorporation of Zn^2+^ is to be expected.

Constant release of Si (silicates) corresponds to the degradation of MBG particles. Since released concentration of silicon was slightly higher in the case of 3–6 composites; it suggested that MBG was degraded probably a bit faster in less stiff composites. Surprisingly, the released concentration of silicates from 0ZnMBG bulk glass and composites was similar and sometimes even a bit lower than from Zn-containing samples even though the starting amount was the same for all MBG. An explanation for this could be that Zn-oxide could switch its role of network modifier and act as network former (along with Si) [61]. From our own non-published experience, it was observed that, depending on the pH, the degradation of MBG is faster with lower buffer capacity, which could explain a bit faster degradation of MBG embedded in hydrogels in composite constructs, showed by release profile of Si. However, limited attention had been paid to the degradation mechanism of bioactive glasses in hydrogels, as mentioned in a previous study about alginate-based bioinks [30]. Concentration of released zinc was similar for bulk glasses and composite scaffolds and it appeared to be quite constant over time. The release of zinc was increased with increasing the starting concentration in samples, as expected, but the release profile from the bulk samples show higher amounts of Ca^2+^ than Zn^2+^ in respective samples containing the same concentration of these ions (0ZnMBG vs. 15ZnMBG) indicating faster release of Ca^2+^ than Zn^2+^. Again, the explanation could be in Zn acting partially as MBG network builder. As expected, released concentration of this ion was directly related to the initial amount of Zn in MBG.

## 5. Conclusions

Calcium was successfully substituted with Zn without affecting the mesoporous structure of the MBG. The inclusion of MBG in algMC ink greatly increased viscosity and appeared to induce a pre-crosslinking effect of alginate. However, substitution of Ca in MBG with Zn mitigated this effect, strongly reducing the viscosity of composite inks. Addition of 7% of MBG containing Ca and/or Zn did not appear to have a negative effect on MSC number development within bioprinted composite constructs, but the ratio of these ions strongly affected the stability of constructs over time. AlgMC blend affected ion release from composites, but released concentrations of bioactive ions were directly dependent on their initial amount in synthesized MBG, making this complex system promising for tailorable composite development.

## Figures and Tables

**Table 1:** Preparation and content of different MBG.

MBG Name	Si mol%	TEOS Grams	P mol%	TEP Grams	Zn mol%	Zn(NO_3_)_2_•6 H_2_O Grams	Ca mol%	Ca(NO_3_)_2_•4 H_2_O Grams
0ZnMBG	80	6.7	5	0.73	0	0	15	1.4
5ZnMBG	80	6.7	5	0.73	5	0.5879	10	0.9333
10ZnMBG	80	6.7	5	0.73	10	1.1757	5	0.4667
15ZnMBG	80	6.7	5	0.73	15	1.7636	0	0

**Table 2:** Three-dimensional printing parameters of composite inks containing 7% of MBG.

algMC Blend	MBG	Pressure [kPa]	Speed [mm/s]
3–6	0ZnMBG	145	4
	5ZnMBG	135	4
	10ZnMBG	70	10
	15ZnMBG	50	14
3–9	0ZnMBG	170	4
	5ZnMBG	230	4
	10ZnMBG	185	4
	15ZnMBG	135	4.5

**Table 3:** Three-dimensional printing parameters of composite bioinks containing 7% of MBG and hTERT-MSC cells.

algMC Blend	MBG	Pressure [kPa]	Speed [mm/s]
3–6	0ZnMBG	130	8
	5ZnMBG	120	8
	10ZnMBG	70	8
	15ZnMBG	40	10
3–9	0ZnMBG	300	4
	5ZnMBG	280	4
	10ZnMBG	165	4
	15ZnMBG	105	5

## Data Availability

The data presented in this study are available on request from the corresponding author.
